# Prilocaine-Induced Methemoglobinemia During Surgically Assisted Rapid Palatal Expansion (SARPE) Under General Anesthesia: A Case Report

**DOI:** 10.1155/crid/9917154

**Published:** 2025-05-09

**Authors:** Yusuke Kurosawa, Karen Gomi, Akinori Moroi, Kunio Yoshizawa, Koichiro Ueki

**Affiliations:** Department of Oral and Maxillofacial Surgery, Division of Clinical Medicine, Interdisciplinary Graduate School, University of Yamanashi, Chuo, Yamanashi, Japan

**Keywords:** local anesthetic, methemoglobinemia, orthognathic surgery, prilocaine

## Abstract

Methemoglobinemia is a condition in which methemoglobin levels in the blood increase. Methemoglobin lacks oxygen-binding ability, resulting in oxygen deprivation in tissues. This high level of methemoglobin in the blood presents as a severely worsening condition. Herein, we report a case of methemoglobinemia caused by the administration of 3% prilocaine hydrochloride containing 0.03 IU/mL feripressin as a local anesthetic during orthognathic surgery. Following drug administration, the patient's atrial oxygen saturation (SpO_2_) decreased. We diagnosed methemoglobinemia based on arterial blood gas analysis, which revealed an increased level of methemoglobin. To maintain the SpO_2_ level, oxygen was administered. The patient's condition improved over time. Careful monitoring and maintenance of SpO_2_ levels are essential to ensure proper diagnosis and effective management.

## 1. Introduction

Methemoglobinemia is a condition in which methemoglobin (hemoglobin in which the Fe^2+^ is oxidized to Fe^3+^) is created [1], meaning that the percentage of methemoglobin in the blood is increased by ≥ 1%–2% [2]. Methemoglobin, which has no oxygen-binding ability, results in a lack of oxygen in tissues. Methemoglobinemia can be classified into two main types: congenital, in which oxygen deficiency or abnormalities occur genetically, and secondary, caused by drugs or other factors [3]. In maxillofacial surgery, secondary methemoglobinemia is reportedly caused by amide-type local anesthetics, such as prilocaine hydrochloride, and ester-type surface anesthetics. Herein, we report a case of methemoglobinemia secondary to the administration of 3% prilocaine hydrochloride (Citanest–Octapressin) containing 0.03 IU/mL feripressin, which was used during orthognathic surgery under general anesthesia.

## 2. Case Presentation

**Figure 1:**
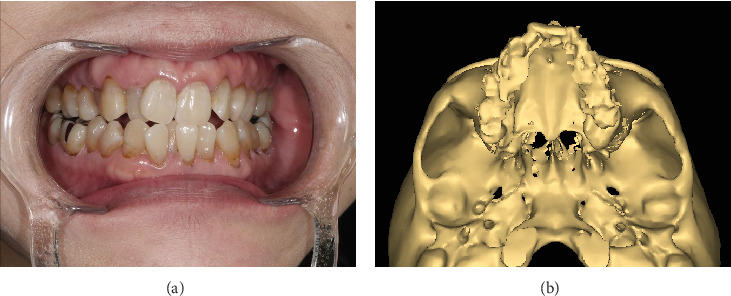
Preoperative photograph and three-dimensional computed tomography results (a and b), showing stenosis of the maxillary dental arch.

A 54-year-old woman was diagnosed with a jaw deformity (Figure 1a,b). Her height and weight were 165.5 cm and 55 kg, respectively. She underwent surgically assisted rapid palatal expansion (SARPE) under general anesthesia.

**Figure 2:**
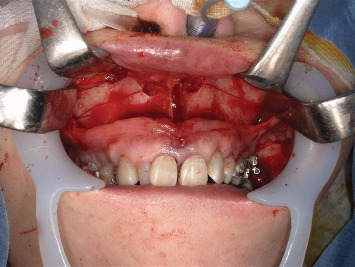
SARPE. The maxillofacial surgeon performs a vertical osteotomy in the maxillary midline, as well as a Le Fort I osteotomy to facilitate expansion.

**Figure 3:**
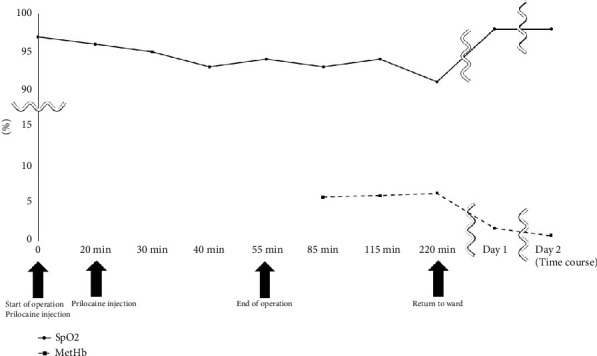
Progressive recordings of oxygen saturation (SpO_2_%) and methemoglobin (MetHb%).

General anesthesia was induced using remifentanil, fentanyl, propofol, and rocuronium. Local anesthesia comprised 3% prilocaine hydrochloride (Citanest–Octapressin) containing 0.03 IU/mL feripressin. She had a history of an allergy to lidocaine (Xylocaine). At the beginning of surgery, 7.2 mL (216 mg prilocaine hydrochloride) of local anesthesia was administered to the gingiva for SARPE and right maxillary wisdom tooth extraction. Twenty minutes following the first administration of local anesthesia, 7.2 mL (216 mg prilocaine hydrochloride) local anesthesia was added to the left mandibular wisdom tooth, right mandibular second molar, and bilateral mandibular first premolar extractions. The operation performed a vertical osteotomy in the maxillary midline, as well as a Le Fort I osteotomy (Figure 2). The SpO_2_ level at the beginning of surgery was 98%. At the end of surgery, the SpO_2_ level decreased to 94% (Figure 3). The operation time was 55 min. Considering the low SpO_2_ at the end of surgery and the risk of nasal hemorrhage and airway obstruction due to maxillary osteotomy, the patient was maintained under intubation to maintain the airway. Chest radiography revealed no abnormal findings. No significant bleeding was observed in the surgical field, and no swelling was observed from the pharynx to the airway. Arterial blood oxygen analysis was performed 30 min after surgery. The methemoglobin level was 5.8%. The patient was diagnosed with methemoglobinemia. After confirming no abnormal findings at the surgical site, the patient was extubated, oxygen was administered at 6 L/min, and the SpO_2_ level was maintained at 94%. As no subjective symptoms such as respiratory distress were observed, the patient was returned to the ward. Subsequently, although oxygen administration was continued, frequent decreases in SpO_2_ were observed. The oxygen concentration was increased to 8 L/min to maintain SpO_2_. Her methemoglobin level was 6.3% at 3 h postoperatively. Thereafter, there was no decrease in SpO_2_ when oxygen was administered, and consciousness was confirmed to have improved.

**Figure 4:**
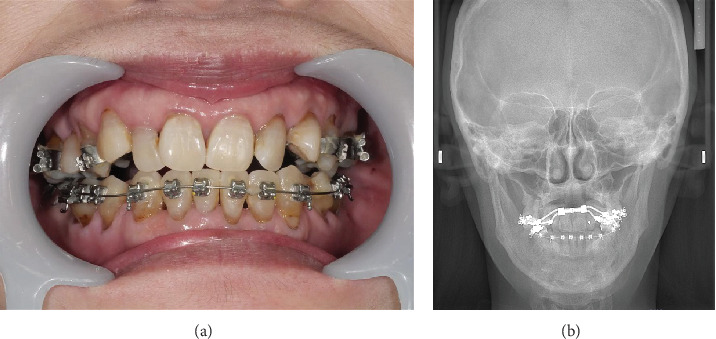
Findings from the postoperative radiograph and photograph show that the stenosis of the maxillary dental arch has improved (a and b).

On Postoperative Day 1, the patient's SpO_2_ and methemoglobin concentrations were 98% and 1.7%, respectively. Oxygen administration was gradually decreased by 1 L/h and was finally completed based on the absence of a decrease in SpO_2_ levels. No decrease in SpO_2_ levels was observed on the following days, and the patient was discharged 7 days postoperatively. Six months after surgery, the patient was doing well. Also, the stenosis of the maxillary dental arch has improved (Figure 4a,b).

## 3. Discussion

Local anesthetics containing vasoconstrictors are the agents of choice during maxillofacial surgery [2]; for example, include lidocaine combined with adrenaline and prilocaine combined with feripressin. Prilocaine is used as a local anesthetic during dental procedures. The reasons for this include the patient's medical history and drug interactions. In the present case, prilocaine was administered because of the patient's history of an allergy to lidocaine. The maximum dose of prilocaine hydrochloride which can be used is 8–9 mg/kg [3]. The patient weighed 55 kg. Therefore, the maximum dose of prilocaine hydrochloride to be administered in this case was 440–495 mg. A dose of 432 mg, which is close to the maximum dose, was used because of the SARPE and four-tooth extractions. Vasters et al. reported that methemoglobin levels range from 0.9% to 15.4% when 300–400 mg prilocaine hydrochloride was used for peripheral nerve block, with large individual differences [4]. In the present case, the drug was administered several times. A large amount of prilocaine hydrochloride in the blood was considered to have caused a marked decrease in the SpO_2_ concentration. When local anesthesia is used frequently, it is necessary to administer a small amount of the drug slowly to check the patient's progress and to administer the drug after confirming that there is no abnormality on the monitor.

In the present case, surgery was performed only on the maxilla, and the duration of the surgery was approximately 1 h. After completing the scheduled procedure, the blood SpO_2_ levels decreased, and we checked for abnormal bleeding and airway stenosis. In cases of prolonged surgery, it is necessary to respond to the symptoms and make a diagnosis while the surgery is ongoing. Maxillofacial surgery commonly involves complex manipulation of structures within the oral cavity, pharynx, and facial skeleton, which can have a significant impact on the airway [5]. Bleeding from nonbright-field sites should be considered, particularly in maxillofacial surgery procedures, such as Le Fort Type I osteotomy. When unexpected symptoms occur, continuous monitoring and early examinations should be performed to ensure safe treatment.

Methemoglobinemia can present as various symptoms, depending on the methemoglobin concentration. Guay and Ryu and Haruhisa reported cyanosis in ≥ 5%, tachycardia in ≥ 10%, and hyperventilation in ≥ 11.5% of cases [6, 7]. Treatment includes continuous oxygenation and the administration of methylene blue or ascorbic acid [2]. Arterial blood oxygen analysis is effective in the diagnosis of methemoglobinemia. Therefore, when there are no abnormalities in the anesthetic devices or maxillofacial region and a decrease in SpO_2_ level is observed, methemoglobinemia should be suspected, and arterial blood oxygen analysis should be performed for an early diagnosis. Since the patient's methemoglobin concentration was not high (maximum 6.3%), methylene blue was not administered. Instead, we administered oxygen to maintain SpO_2_ levels. During follow-up, methemoglobin levels were decreased by methemoglobin reductase, and the patient was in spontaneous remission. In case of respiratory deterioration and ventilatory disturbance, methylene blue and ascorbic acid should be considered at an early stage [2].

Careful monitoring and maintenance of SpO_2_ levels are essential to ensure proper diagnosis and effective management of patients during surgery.

## Data Availability

All data related to the present case are included in the published article.
